# Sequence Based Prediction of Antioxidant Proteins Using a Classifier Selection Strategy

**DOI:** 10.1371/journal.pone.0163274

**Published:** 2016-09-23

**Authors:** Lina Zhang, Chengjin Zhang, Rui Gao, Runtao Yang, Qing Song

**Affiliations:** 1 School of Control Science and Engineering, Shandong University, Jinan, China; 2 School of Mechanical, Electrical and Information Engineering, Shandong University at Weihai, China; 3 School of Electrical Engineering, University of Jinan, Jinan, China; Weizmann Institute of Science, ISRAEL

## Abstract

Antioxidant proteins perform significant functions in maintaining oxidation/antioxidation balance and have potential therapies for some diseases. Accurate identification of antioxidant proteins could contribute to revealing physiological processes of oxidation/antioxidation balance and developing novel antioxidation-based drugs. In this study, an ensemble method is presented to predict antioxidant proteins with hybrid features, incorporating SSI (Secondary Structure Information), PSSM (Position Specific Scoring Matrix), RSA (Relative Solvent Accessibility), and CTD (Composition, Transition, Distribution). The prediction results of the ensemble predictor are determined by an average of prediction results of multiple base classifiers. Based on a classifier selection strategy, we obtain an optimal ensemble classifier composed of RF (Random Forest), SMO (Sequential Minimal Optimization), NNA (Nearest Neighbor Algorithm), and J48 with an accuracy of 0.925. A Relief combined with IFS (Incremental Feature Selection) method is adopted to obtain optimal features from hybrid features. With the optimal features, the ensemble method achieves improved performance with a sensitivity of 0.95, a specificity of 0.93, an accuracy of 0.94, and an MCC (Matthew’s Correlation Coefficient) of 0.880, far better than the existing method. To evaluate the prediction performance objectively, the proposed method is compared with existing methods on the same independent testing dataset. Encouragingly, our method performs better than previous studies. In addition, our method achieves more balanced performance with a sensitivity of 0.878 and a specificity of 0.860. These results suggest that the proposed ensemble method can be a potential candidate for antioxidant protein prediction. For public access, we develop a user-friendly web server for antioxidant protein identification that is freely accessible at http://antioxidant.weka.cc.

## 1 Introduction

ROS (Reactive Oxygen Species) are generated in aerobic metabolic processes as a result of endogenous and exogenous factors, such as air pollutants and cigarette smoke [1]. Moderate concentrations of ROS can function in physiological oxidative processes of cells [2, 3], including gene expression and signal transduction [4]. However, high concentrations of ROS are considered to be harmful to cells, which can naturally produce excess oxygen free radicals beyond the ability of various antioxidants to eliminate or detoxify their harmful effects [5], thereby giving rise to oxidative stress.

Oxidative stress can damage cell constituents via injury to macro molecules such as carbohydrates, DNA, and proteins [6, 7]. Consequently, it has been recognized that the oxidative stress is associated with the pathogenesis of many diseases, including cancers, cataractogenesis, atherosclerosis, neurodegenerative diseases, diabetes, autism spectrum disorder, down syndrome, and asthma [8–10]. In addition, oxidative stress is thought to be involved in aging problems [11]. In the food industry, oxidative stress has been indicated to be closely related with beer aging [12]. It is well known that oxidative stress cause deteriorations of food quality and shortening of shelf life [13, 14]. Therefore, to protect against such ROS-induced damages, it is critical to maintain a balance between oxidative and antioxidative process with help of antioxidative protection, which plays a dominant role in cell viability, activation, proliferation, and organ function [1].

Antioxidants can quench oxygen free radical reactions by interacting with and neutralizing free radicals, which is absolutely critical for maintaining the body’s redox balance [15], protecting beer against aging, and avoiding food deteriorations [12]. A variety of artificial antioxidants exhibit strong antioxidant activity. However, they are restricted in many fields due to their potential health risks [16]. Therefore, identification of effective natural antioxidants is of great interest [3, 14].

Antioxidant proteins have a great potential to prevent or slow the progression of some diseases, such as some DNA-induced diseases [17], reperfusion injury, traumatic brain injury [18], and cancers [19]. Antioxidant proteins are implicated in natural life-span [20] due to the ability to eliminate aging damage caused by oxidative stress. In addition, they contribute to the endogenous antioxidant capacity of foods to maintain the food texture and color. In view of the powerful functions of antioxidant proteins to provide protection against serious diseases and prevent foods from undergoing deteriorations, accurately identifying antioxidant proteins could provide useful clues to reveal physiological processes of certain types of diseases, aging and food deteriorations, thereby providing a rational basis for developing novel antioxidation-based drugs that can cure or alleviate these types of diseases, slow down the aging process and extend food shelf-life.

Identification of antioxidant proteins through traditional experimental methods is time-consuming and laborious. It is in great need to develop computational methods. Despite its importance, few computational methods have been proposed. Feng PM et al. [21] carried out a Naïve Bayes-based predictor using amino acid composition and dipeptide composition. Later on, our group [22] investigated the performance of g-gap dipeptide composition and PSSM (Position Specific Scoring Matrix) on a RF (Random Forest) classifier. We demonstrated that g-gap dipeptide composition could be appropriate feature descriptors for this classification problem. Although the existing methods have their own merits, they still have some shortcomings to address. (1) The method proposed in [21] extracted the correlations between two adjoining amino acids of protein sequences using dipeptide composition without incorporating higher tier correlations of residues, which may affect the prediction quality. The method proposed in [22] used g-gap dipeptide composition to search for the important correlations between two residues. However, the distribution information of protein sequences is missing. (2) Singularity of feature extraction strategies is an unignorable fact in previous methods. Some useful features which can reflect the properties of antioxidant proteins may be lost. Generally, multiple feature extraction strategies can complement each other to extract valuable information from various sources, which is critical to improve the performance and robustness of a predictor [23, 24]. (3) Previous methods both developed a predictor based on an individual classifier. An individual classifier usually has its own inherent defects, which would result in poor prediction performance [25]. Ensemble classifier integrates diversity learning strategies of multiple individual classifiers, which can perform better than its component individual classifiers in protein attribution prediction [26].

**Fig 1:**
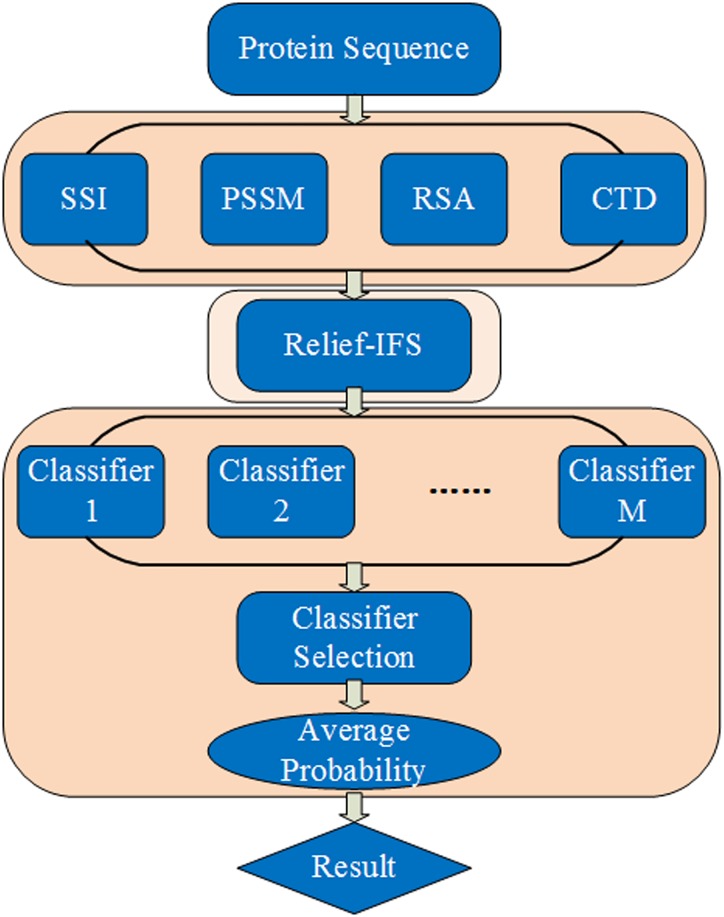
The computational framework of the proposed predictor. SSI: Secondary Structure Information; PSSM: Position Specific Scoring Matrix; RSA: Relative Solvent Accessibility; CTD: Composition, Transition, Distribution; IFS: Incremental Feature Selection.

To address the above-mentioned limitations and improve prediction performance with respect to antioxidant proteins, we propose an ensemble predictor using a classifier selection strategy with hybrid features, including SSI (Secondary Structure Information), PSSM (Position Specific Scoring Matrix), RSA (Relative Solvent Accessibility), and CTD (Composition, Transition, Distribution). The Relief combined with IFS (Incremental Feature Selection) method is used to select high discriminative features for reducing the computational complexity and improving prediction capability. The prediction results of the ensemble predictor are determined by an average of prediction results of multiple base classifiers. The computational framework of the proposed predictor is illustrated in Fig 1. To evaluate the performance of our ensemble predictor objectively, the present model is compared with [21, 22] based on the same independent testing dataset.

## 2 Materials and Methods

### 2.1 Data Collection

To facilitate comparisons with previous studies in identifying antioxidant proteins, we use the benchmark dataset constructed in [22]. Only those protein sequences from the UniProtKB/Swiss-Prot database [27] reviewed and annotated by antioxidant in the molecular function of gene ontology, are selected. In order to obtain the reliable dataset, the following criteria are further performed. (1) Sequences which are fragments of other proteins are excluded because their information is redundant and not integrity. (2) Sequences containing nonstandard letters except 20 standard amino acid alphabets are removed because their meanings are ambiguous.

After the above screening procedures, 482 antioxidant protein sequences are obtained as the original positive dataset. Due to the number of non-antioxidant protein sequences is extremely large, 500 non-antioxidant protein sequences are randomly selected as the original negative dataset. In order to avoid over fitting problem, none of the sequences has ≥70% sequence identity to any other in the original dataset by means of CD-HIT program [28]. The final benchmark dataset consists of 174 antioxidant proteins and 492 non-antioxidant proteins. In order to validate the performance of our proposed predictor objectively, 100 antioxidant and 100 non-antioxidant proteins are respectively selected from the final benchmark dataset as the training dataset and the rest with 74 antioxidant and 392 non-antioxidant proteins as the independent testing dataset. The samples in the independent testing dataset are not in the training dataset. The benchmark dataset is available in S1 Table.

### 2.2 Feature Extraction

To develop high throughput tools for predicting complicated protein attributes, it is important to represent a protein sequence with a comprehensive and proper feature vector with a fixed length [29]. In general, an individual feature extraction strategy can only preserve partial target’s knowledge, thereby limiting prediction performance. Multiple feature representation methods from different sources can be complementary in capturing valuable information to enhance the discrimination power of a hypothesis. In this study, we employ hybrid features extracted from SSI, PSSM, RSA, and CTD to represent antioxidant proteins.

#### 2.2.1 Secondary Structure Information

Protein secondary structure determines most protein reactions and reveals the intricate function of protein sequences to a great extent [30, 31]. The contents and spatial arrangements of secondary structure elements are significant factors that influence the protein intricate functions or structures [32]. The Porter 4.0 server [33] is used in this study to predict three-state secondary structures. It predicts every amino acid in a protein sequence into one of the three secondary structure elements, i.e. *H* (helix), *E* (strand), and *C* (coil). The following related features are extracted from the secondary structure elements.

(i) Content information is one of the most widely used secondary structure features [32], which is defined as (3 features)

$$\begin{array}{c} F_{j}=\frac{N_{j}}{L},j=\left\{H,E,C\right\}, \end{array}$$ (1)

where *N*_*j*_ is the number of helix/strand/coil element and *L* is the length of the protein sequence.

(ii) Transition information of helix/strand/coil along a protein sequence is calculated by the following equation. (9 features)

$$\begin{array}{c} T_{i,j}=\frac{N_{i,j}}{L-1},i,j=\left\{H,E,C\right\}, \end{array}$$ (2)

where *N*_*i*, *j*_ denotes the number of secondary structure element combinations from the secondary structure type of helix/strand/coil.

(iii) The average length and the normalized maximal length of the segments with each secondary structure type are calculated as (6 features)

$$\begin{array}{c} AvgSeg_{i}=\frac{\sum Len(Seg_{i})}{\sum Seg_{i}},i=\left\{H,E,C\right\}, \end{array}$$ (3)

$$\begin{array}{c} NMaxSeg_{i}=\frac{Max(Seg_{i})}{L},i=\left\{H,E,C\right\}, \end{array}$$ (4)

where *Seg*_*i*_ denotes the segment composed of secondary structure element helix/strand/coil. *Len*(*Seg*_*i*_) is the length of *Seg*_*i*_. *Max* is the maximal function of segment length.

(iv) Order-related features from secondary structure elements are introduced to reflect the special arrangements of the secondary structure elements, which are formulated as (3 features)

$$\begin{array}{c} F_{i}=\sum_{j=1}^{N_{i}}p_{i,j}/\;L(L-1),i=\left\{H,E,C\right\}, \end{array}$$ (5)

where *N*_*i*_ is the number of helix/strand/coil element. *p*_*i*, *j*_ denotes the position of the *j*th order of the corresponding secondary structure element.

#### 2.2.2 Position Specific Scoring Matrix

With the avalanche of genome sequences generated in the post-genomic age, the completed human genome provides a large number of novel proteins containing conserved domains [34]. The conserved domains serve as evidence for structural and functional conservations [35]. Evolutionary conservations can determine important biological functions and are important in biological sequence analysis [36]. The PSSM (Position Specific Score Matrix) is adopted here to obtain the evolutionary conservations and some essential signatures of protein sequences, which has been widely employed in protein attribute prediction problems [37, 38]. The PSSM is a matrix of score values, which is derived from the PSI-BLAST (Position-Specific Iterative Basic Local Alignment Search Tool) [39] with 3 iterations and the E-value cutoff of 0.0001.

For a given protein sequence with *L* amino acids, the corresponding PSSM has L*20 elements, and is defined as

$$\begin{array}{c} P_{\text{PSSM}}=\left[\begin{array}{cccccc} E_{1\to 1} & E_{1\to 2} & \cdots & E_{1\to j} & \cdots & E_{1\to 20}\\ E_{2\to 1} & E_{2\to 2} & \cdots & E_{2\to j} & \cdots & E_{2\to 20}\\ \vdots & \vdots & \cdots & \vdots & \cdots & \vdots \\ E_{i\to 1} & E_{i\to 2} & \cdots & E_{i\to j} & \cdots & E_{i\to 20}\\ \vdots & \vdots & \cdots & \vdots & \cdots & \vdots \\ E_{L\to 1} & E_{L\to 2} & \cdots & E_{L\to j} & \cdots & E_{L\to 20} \end{array}\right], \end{array}$$ (6)

where the rows and columns of the PSSM are indexed by the protein residues and 20 native amino acids, respectively. The values in the *i*th row denote the probabilities of the *i*th residue of the given protein sequence mutating to 20 native amino acids during the evolution process.

To formulate protein sequences into the feature vectors with the same dimension, all the rows in the PSSM corresponding to the same amino acids in a protein sequence are summed up. Then the PSSM is transformed into a 20 × 20 dimensional matrix. These 400 elements are extracted from the PSSM to encode protein sequences.

#### 2.2.3 Relative Solvent Accessibility

Solvent accessibility is a key property of amino acid residues and plays an important part in a protein’s function [40]. The accessible surface area of a protein is closely related with its overall antioxidant activity. More solvent accessibility of amino acid residues represents high antioxidant activity of a protein, due to the fact that free radicals and chelate prooxidative metals can be scavenged [13]. Therefore, it is reasonable to extract features from RSA (Relative Solvent Accessibility).

The RSA is defined as the solvent ASA (Accessible Surface Area) of a given residue normalized by the ASA of this residue in an extended tripeptide, Ala-X-Ala, conformation [41]. The RSA values are predicted by PaleAle 4.0 [33]. Using the software, each residue of the query sequence is assigned a buried or exposed state.

The following 28 features are designed to encode each protein sequence. (i) Mean/standard deviation of all residues’ RSA scores (2 features). (ii) Number of buried/exposed segments (2 features). (iii) Minimum/maximum length of buried/exposed segments (4 features). (iv) Average RSA score of each native amino acid (20 features).

#### 2.2.4 Composition, Transition, Distribution

**Fig 2:**
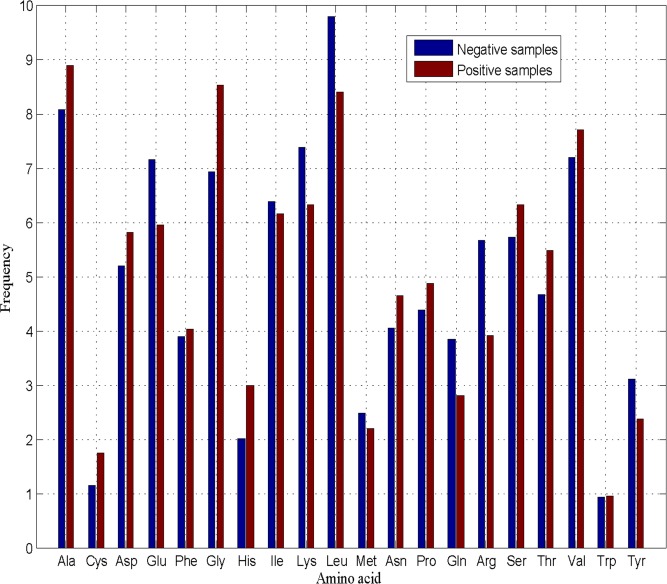
Amino acid composition analysis of the residues in antioxidant proteins and non-antioxidant proteins. We analyze amino acid composition of the residues in positive samples and negative samples. There is a big difference in terms of amino acid compositions between positive samples and negative samples.

We analyze amino acid composition of the residues in positive samples and negative samples. As shown in Fig 2, there is a big difference in terms of amino acid compositions between positive samples and negative samples. To further extract information on composition, order, and distribution from protein sequences, a global feature extraction strategy called CTD (Composition, Transition, Distribution), introduced by Dubchak et al. [42], is adopted to encode protein sequences.

Using CTD, three global descriptors, composition (C), transition (T) and distribution (D) are employed in this study to describe the properties of protein sequences. For a given protein sequence, composition (C) describes the global percent composition of 20 native amino acids (20 features). Transition (T) characterizes the percent frequency with amino acids of one type of native amino acids followed by another type (190 features). Distribution (D) measures the respective locations of the first, 25%, 50%, 75% and 100% of each type of 20 native amino acids (100 features). For detailed description about the CTD method, please refer to [42].

### 2.3 Feature Selection

After carrying out feature extraction strategies mentioned above, protein sequences are formulated by numerical feature vectors with the same dimension. However, they may not contribute equally to identifying antioxidant proteins on account of redundant and irrelevant features. These additional features may deteriorate performance of a classifier, slow down the learning process and decrease the generalization power of the learned classifiers [43]. Feature selection is an effective way to overcome these disadvantages, which can contribute to improving the classification accuracy of a classifier, simplifying a classifier, and thereby better understanding the potential physical meaning in data [44]. In this study, Relief-IFS is adopted to search the optimal features.

**Relief**. The Relief algorithm, originally proposed by Kira [45], is a feature-weighting algorithm, which is considered one of the most successful algorithms for depicting the relevance between the features and class labels. It is noise-tolerant and requires only linear time. Based on the ability of the feature to distinguish the near samples, the Relief algorithm can be used to estimate the quality of each feature [46]. The feature with a larger weight indicates a more highly relevant one for the target prediction. The Relief algorithm is executed iteratively. During each iteration process, the Relief algorithm endows each feature with a weight as formulated by

$$\begin{array}{c} W_{p}^{i+1}=W_{p}^{i}-\frac{diff(Y,x_{i},H\left(x_{i}\right))}{m}+\frac{diff(S,x_{i},M\left(x_{i}\right))}{m}, \end{array}$$ (7)

$$\begin{array}{c} diff\left(*,x,y\right)=\left\{\begin{array}{c} \left\|x-y\right\|\;\;\;,\;x\neq y\\ \;\;\;\;\;\;\;\;\;\;\;0,\;\;\;\;\;\;\;\;\;\;\;\;\;\;\;\;\;\;\;\;\;\;\;\;\;\;\;\;\;\;x=y \end{array}\right., \end{array}$$ (8)

where $W_{p}^{i}$ and $W_{p}^{i+1}$ denote the current and next weights, respectively. *p* represents a given feature. *x*_*i*_ stands for the *i*th sample sequence. *H*(*x*_*i*_), termed as the nearest hit, represents the nearest neighbor samples from the same class label against *x*_*i*_. *M*(*x*_*i*_), referred to as the nearest miss, strands for the nearest neighbor samples from the different class labels against *x*_*i*_. *Y* and *S* denote the sample sets with the same and different class labels against *x*_*i*_, respectively. *m* is the number of random samples. The function of *diff*(*, *x*, *y*) is used for calculating the distance between the random samples to find the nearest neighbor.

The ranked feature list can be obtained based on weights, represented as

$$\begin{array}{c} \{f_{1},f_{2},\cdots ,f_{N}\}, \end{array}$$ (9)

where *f*_1_ represents the feature with the highest weight, *f*_2_ with the second highest,⋯, and *f*_*N*_ with the lowest.

**Incremental Feature Selection**. Based on the ranked feature list evaluated by Relief, IFS(Incremental Feature Selection), one of the well-known searching strategies of feature selection, is employed to determine the optimal feature subset. During the IFS procedure, the feature subset starts with one feature with the highest Relief weigh. Then, features in the ranked feature list are added one by one from higher to lower rank into the feature subset [47]. A new feature subset is generated when a new feature from the feature list is added. In this study, individual predictors for all feature subsets are constructed using our ensemble classifier and evaluated by 10-fold cross validation on the training dataset. The feature subset that has the highest accuracy is selected as the final input of the optimal ensemble classifier.

The WEKA (Waikato Environment for Knowledge Analysis) software package [48] is used for the feature selection algorithm Relief, where default parameters are employed. The software package can be downloaded at http://www.cs.waikato.ac.nz/ml/weka/downloading.html.

### 2.4 Ensemble Learning Method

Every single classifier usually has its own inherent defects, and it could not always perform well on all datasets [49]. Generally, a well-defined ensemble classifier is able to address statistical, computational, and representational issues better than its component individual classifiers [50] due to the fact that ensemble classifier is able to make use of the different decision boundaries generated from the individual classifiers to strategically combine the classification results [51, 52]. The prediction performance of an ensemble classifier is affected by diversity and individual accuracy of its component base classifiers [53, 54].

To achieve satisfactory prediction results, we use an ensemble of different individual classifiers for antioxidant protein prediction. Firstly, we choose 10 different base classifiers, including RF (Random Forest), SMO (Sequential Minimal Optimization), NNA (Nearest Neighbor Algorithm), J48, BN (BayesNet), RBFNetwork, DT (Decision Table), Adaboost, VFI, and NB (Naïve Bayes).

These 10 base classifiers are trained and ranked according to the accuracy. A ranked classifier list is obtained and represented as

$$\begin{array}{c} \{C_{1},C_{2},\cdots ,C_{i},\cdots ,C_{10}\}, \end{array}$$ (10)

where *C*_1_ represents a classifier with the highest accuracy, *C*_2_ with the second highest,⋯, and *C*_10_ with the lowest.

**Fig 3:**
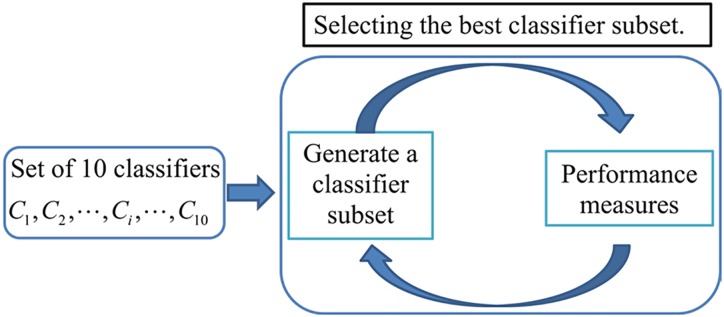
Diagram of the classifier selection method. Classifier subset starts with one classifier with the highest accuracy. Then, classifiers in the ranked classifier list are added one by one from higher to lower rank into the classifier subset. We evaluate prediction performance of each classifier subset. The classifier subset with the highest accuracy is selected to construct the ensemble predictor.

Based on the ranked classifier list, the idea of IFS is employed to determine the optimal classifier subset. Classifier subset starts with one classifier with the highest accuracy. Then, classifiers in the ranked classifier list are added one by one from higher to lower rank into the classifier subset. A new classifier subset is generated when a new classifier is added. We evaluate prediction performance of each classifier subset. The classifier subset with the highest accuracy is selected to construct the ensemble predictor. The prediction results of each base classifier in the selected classifier subset are combined using average probability. Fig 3 shows the diagram of the classifier selection method.

### 2.5 Performance Measures

In statistical prediction, there are 3 cross-validation methods often used to examine the accuracy, i.e. independent dataset test, sub-sampling test (e.g. 5-fold or 10-fold cross validation), and jackknife test [55]. Among these three methods, the jackknife test is deemed the most objective and rigorous one that can exclude the memory effects during the entire testing process and can always yield a unique result for a given benchmark dataset, as elucidated in [56] and demonstrated by Eq 50 of Chou and Shen [57]. Therefore, the jackknife test has been increasingly and widely adopted by investigators to test the power of various predictors [58, 59]. To reduce the computational complexity, 10-fold cross validation test is employed in this paper. During the procedure, the training dataset is randomly separated into 10 equally-sized parts. Each time, 9 parts are merged as training dataset to train a model, and then the other one part is for testing the model. This process is repeated ten times to test each part. The ultimate result is the average of the 10 prediction results. To assess performance of the predictor intuitively, 4 most common used indexes are employed.

Sensitivity (*Sn*) is the percentage of correctly identified antioxidant proteins and given by

$$\begin{array}{c} S_{n}=\frac{TP}{TP+FN}, \end{array}$$ (11)

Specificity (*Sp*) is the percentage of correctly identified non-antioxidant proteins and defined as

$$\begin{array}{c} S_{p}=\frac{TN}{TN+FP}, \end{array}$$ (12)

Accuracy (*Acc*) is the percentage of correctly identified antioxidant proteins and non-antioxidant proteins and expressed as

$$\begin{array}{c} Acc=\frac{TP+TN}{TP+FP+TN+FN}, \end{array}$$ (13)

MCC (Matthew’s Correlation Coefficient) is a more stringent measure of prediction accuracy accounting for both under and over-predictions [60], which is given by

$$\begin{array}{c} MCC=\frac{TP*TN-FP*FN}{\sqrt{\left(TP+FN)(TP+FP)(TN+FP)(TN+FN\right)}}, \end{array}$$ (14)

where *TP*, *FP*, *TN*, and *FN* represent true positive, false positive, true negative, and false negative, respectively.

To further evaluate performance of a predictor, the ROC (Receiver Operating Characteristic) curve is also employed [61]. The ROC curve is plotted with the *Sn* as the *y*-axis and 1 − *Sp* as the *x*-axis by varying the thresholds. The AUC (Area Under the ROC Curve) is a valid measure used for model evaluation obtained from the ROC curve. The higher AUC value corresponds to better performance of a predictor.

## 3 Results and Discussion

### 3.1 Performance Comparisons of Various Individual Classifiers

We select 10 base classifiers and test prediction performance of these individual classifiers. Table 1 shows performance comparisons of these individual classifiers on the training dataset by 10-fold cross validation. From Table 1, the accuracy values of various classifiers are in the range of 0.755 to 0.895, much better than random guess (i.e., an accuracy of 0.500), indicating acceptable performance in antioxidant protein prediction. Among the various individual classifiers, RF achieves the best performance with an accuracy of 0.895, an MCC of 0.790, and an AUC of 0.957, followed by SMO, NNA, J48, BN, RBFNetwork, DT, Adaboost, VFI, and NB. In addition, RF obtains balanced performance with an Sn of 0.9 and an Sp of 0.89. These results demonstrate that RF is relatively effective in antioxidant protein identification.

**Table 1:** Performance comparisons of multiple individual classifiers on the training dataset by 10-fold cross validation.

Classifier	Sn	Sp	Acc	MCC	AUC
RF	0.9	0.89	0.895	0.790	0.957
SMO	0.92	0.85	0.885	0.772	0.885
NNA	0.94	0.79	0.865	0.738	0.865
J48	0.87	0.85	0.86	0.720	0.852
BN	0.92	0.77	0.845	0.698	0.933
RBFNetwork	0.91	0.78	0.845	0.696	0.861
DT	0.84	0.83	0.835	0.670	0.858
Adaboost	0.88	0.78	0.83	0.6635	0.901
VFI	0.74	0.79	0.765	0.531	0.763
NB	0.92	0.59	0.755	0.540	0.888

### 3.2 Performance Comparisons of Ensemble Classifiers

**Fig 4:**
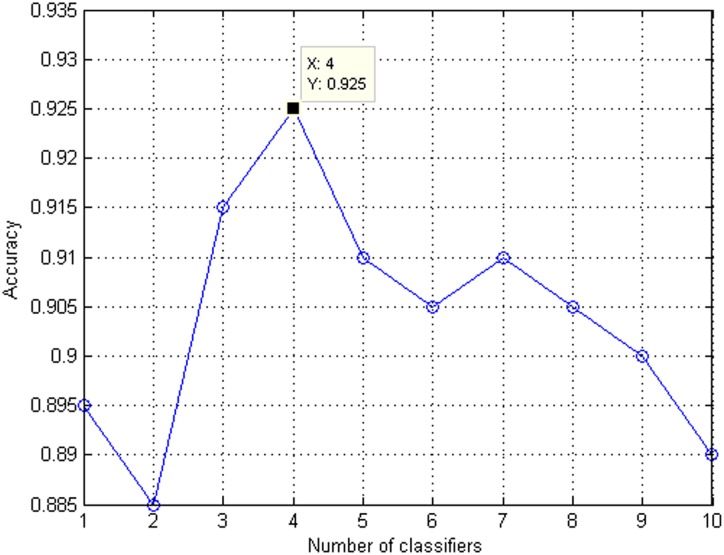
Prediction accuracy against different classifier subsets. Classifier subset starts with one classifier with the highest accuracy. Then, classifiers in the ranked classifier list are added one by one from higher to lower rank into the classifier subset. A new classifier subset is generated when a new classifier is added. We evaluate prediction performance of each classifier subset.

To obtain the optimal ensemble classifier for antioxidant protein identification, we evaluate the accuracy of multiple individual classifiers and get a classifier list ranked by accuracy. In the classifier list, a classifier with a smaller index represents a more important one for antioxidant protein identification. The classifier list is used to select the optimal classifier subset according to the idea of IFS procedure. Add the ranked classifiers one by one from the top of the classifier list to the bottom, then, the predictor is accordingly built for each classifier subset and evaluated on the training dataset by 10-fold cross validation. Prediction performance of classifier subsets is shown in Table 2 and the prediction accuracy values against classifier subsets are depicted in Fig 4.

**Table 2:** Prediction performance of different classifier subsets on the training dataset by 10-fold cross validation.

Ensemble Classifier	Sn	Sp	Acc	MCC	AUC
RF	0.9	0.89	0.895	0.790	0.957
RF+SMO	0.92	0.85	0.885	0.772	0.963
RF+SMO+NNA	0.94	0.89	0.915	0.831	0.963
RF+SMO+NNA+J48	0.94	0.91	0.925	0.850	0.961
RF+SMO+NNA+J48+BN	0.93	0.89	0.91	0.821	0.961
RF+SMO+NNA+J48+BN+RBFNetwork	0.93	0.88	0.905	0.811	0.957
RF+SMO+NNA+J48+BN+RBFNetwork+DT	0.93	0.89	0.91	0.821	0.954
RF+SMO+NNA+J48+BN+RBFNetwork+DT+Adaboost	0.92	0.89	0.905	0.810	0.958
RF+SMO+NNA+J48+BN+RBFNetwork+DT+Adaboost+VFI	0.92	0.88	0.9	0.801	0.953
RF+SMO+NNA+J48+BN+RBFNetwork+DT+Adaboost+VFI+NB	0.92	0.86	0.89	0.781	0.951

From Table 2 and Fig 4, as the number of classifiers increases, accuracy shows an upward trend in the initial phase. Afterwards, accuracy shows a downward trend with the increase of number of classifiers. The best accuracy reaches 0.925 when 4 classifiers are selected, including RF, SMO, NNA, and J48. These 4 classifiers are used to construct the optimal ensemble classifier for predicting antioxidant proteins. The default parameters of these four base classification algorithms in WEKA are used in this paper. This ensemble classifier also achieves the best sensitivity of 0.94, specificity of 0.91, and MCC of 0.850. These results indicate that the ensemble classifier is effective in predicting antioxidant proteins. We should also note that the combination of more base classifiers don’t always achieve better performance due to the fact that these base classifiers may share similar learning strategies more or less.

### 3.3 Performance Comparisons of Ensemble Learning Method and Individual Base Classifiers

To verify the strength of the proposed ensemble method, prediction results of our ensemble method and its component base classifiers, including RF, SMO, NNA, and J48, are compared. From Tables 1 and 2, although NNA obtains an identical sensitivity of 0.94 as the ensemble classifier, it yields the lowest specificity of 0.79. The ensemble classifier achieves much better prediction performance than these 4 base classifiers, indicating that a well-established ensemble classifier can deal with protein function prediction better than its component base classifiers. Except an ensemble classifier composed of 2 classifiers and another one composed of 10 classifiers, the accuracy values of the other 8 ensemble classifiers are all better than those of the corresponding component base classifiers. These results are in accordance with the statement in Subsection ‘Ensemble Learning Method’ that a well-defined ensemble classifier with diversity base classifiers and the reasonable accuracy can address classification issues better than its component individual classifiers.

### 3.4 Feature Selection Results

**Fig 5:**
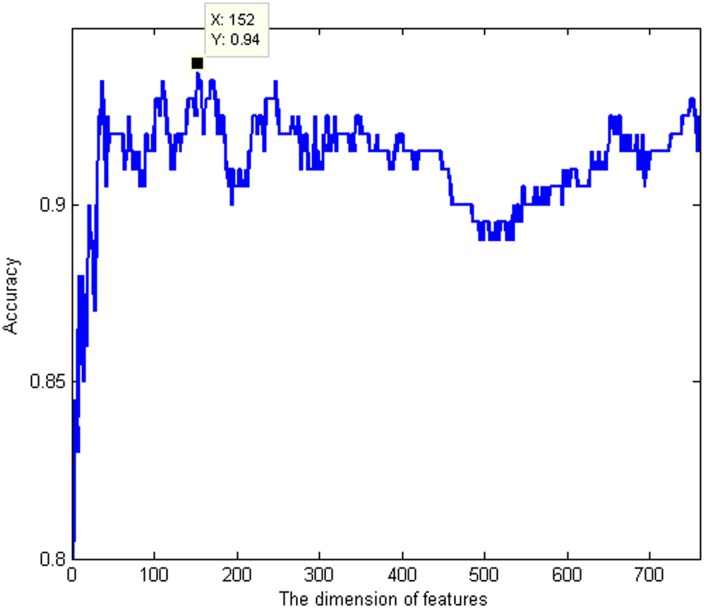
The IFS curve: the values of accuracy against the dimension of features. By adding features one by one from higher to lower rank, 759 different feature subsets are obtained. The individual predictor is then accordingly built for each feature subset and evaluated by 10-fold cross validation. The IFS curve reveals the relation between the accuracy and the feature subsets.

The hybrid features are ranked based on the Relief method. Within the feature list (see S2 Table), a feature with a smaller index represents a more important one for antioxidant protein prediction. Then, the IFS method combined with our ensemble classifier is employed to search the optimal features. In the IFS procedures, adding the ranked features one by one, individual predictors for all the feature subsets are constructed using our ensemble classifier and evaluated by 10-fold cross validation. The IFS results are given in S3 Table. The IFS curve is plotted in Fig 5, which shows the relationship of feature indices and accuracy. From Fig 5, the curve reaches its peak with an accuracy of 0.94, when the first 152 features in the S2 Table are selected. These features are regarded as the optimal features for antioxidant protein prediction.

### 3.5 Contribution of Feature Selection to Our Ensemble Classifier

**Fig 6:**
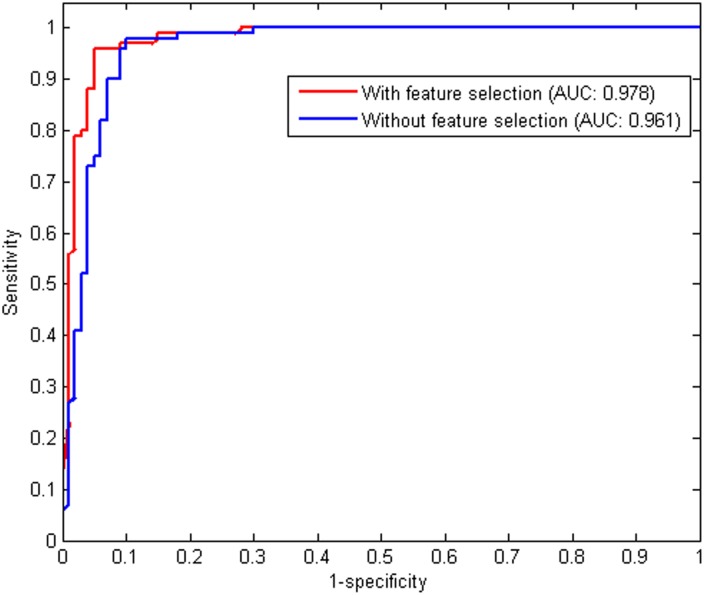
ROC curves of the ensemble classifier with and without feature selection. The ROC curve is plotted with the *Sn* as the *y*-axis and 1 − *Sp* as the *x*-axis by varying the thresholds. The AUC is a valid measure used for model evaluation obtained from the ROC curve. The higher AUC value corresponds to better performance of a predictor.

To investigate the influence of feature selection on the performance of the ensemble classifier, the prediction results of the ensemble method with and without feature selection are shown in Table 3. Fig 6 depicts the ROC curves obtained with and without feature selection. From Table 3 and Fig 6, the ensemble method with feature selection achieves a sensitivity of 0.95, a specificity of 0.93, an accuracy of 0.94, an MCC of 0.880, and an AUC of 0.978, which are all superior to those of the ensemble method without feature selection. These results demonstrate that many redundant or uninformative features are present in the original feature sets and the Relief-IFS method can significantly remove these useless features to greatly improve the performance of the ensemble model. The ensemble classifier with feature selection is determined as the final predictor for antioxidant protein prediction.

**Table 3:** Prediction performance of our ensemble classifier with feature selection or not.

Method	Sn	Sp	Acc	MCC	AUC
Without feature selection	0.94	0.91	0.925	0.850	0.961
With feature selection	0.95	0.93	0.94	0.880	0.978

### 3.6 Analysis of the Optimal Features

**Fig 7:**
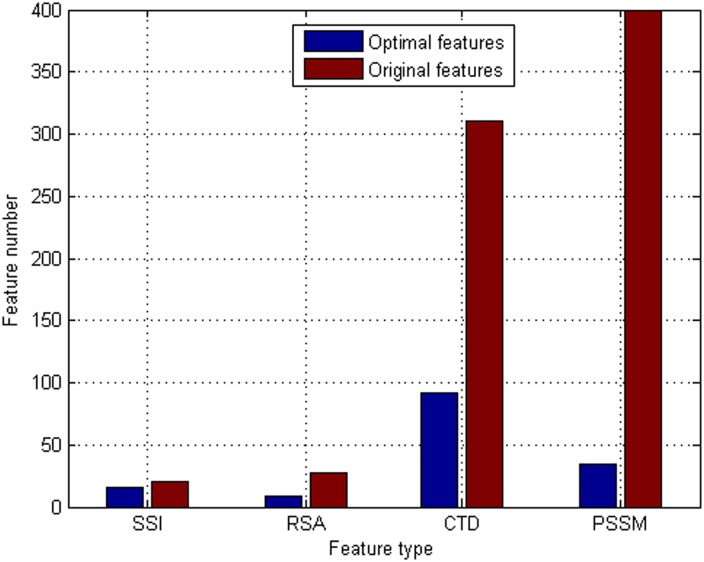
Distribution of each type of features in the optimal features and original features. The optimal prediction model uses the first 152 features in the Relief feature list to encode protein sequences. These 152 features are deemed to be the optimal features for identifying antioxidant proteins. To discover the different contributions of various types of features, the distribution of each type of features in the optimal features and original features is investigated.

The feature type distributions of the original features and the optimal features are investigated and shown in Fig 7. From Fig 7, among the 152 optimal features, there are 16 SSI features, 9 RSA features, 92 DCT features, and 35 PSSM features, indicating that all kinds of features contribute to the prediction of antioxidant proteins.

To evaluate which feature types make more contributions to prediction performance of antioxidant proteins, the percentages of the optimal features accounting for the corresponding feature types are also investigated. The ratios of selected/total features in each feature type are 16/21 = 76.19% (SSI), 9/28 = 32.14% (RSA), 92/310 = 29.68% (CTD), and 35/400 = 8.75% (PSSM), indicating that SSI features play a crucial role in predicting antioxidant proteins. Protein secondary structure reveals the intricate function of protein sequences to a great extent [30, 31]. This is the first attempt to employ SSI based features for antioxidant protein prediction, which may help provide new annotations for the properties of antioxidant proteins. 32.14% of RSA features are selected as the optimal features, indicating that RSA based features play an irreplaceable role in predicting antioxidant proteins. Solvent accessibility plays an important part in a protein’s function [40]. The accessible surface area of a protein is closely related with its overall antioxidant activity. More solvent accessibility of amino acid residues represents high antioxidant activity of a protein, due to the fact that free radicals and chelate prooxidative metals can be scavenged [13]. We analyze amino acid composition of the residues in positive samples and negative samples. There is a big difference in terms of amino acid compositions between positive samples and negative samples. CTD based features account for reasonable proportions of the optimal feature set. This implies that information on composition, transition and distribution plays some roles in predicting antioxidant proteins. It is noted that the ratio of PSSM based features is slightly smaller compared to that of other feature types, due to the fact that the number of this feature type in the original feature set is the most of all those of other feature types. Evolutionary conservations can determine important biological functions [36]. PSSM based features are also necessary in predicting antioxidant proteins.

The proposed predictor is designed through analyzing the sequence characteristics and other characteristics about antioxidant functions of antioxidant proteins to distinguish between antioxidant proteins and non-antioxidant proteins, which can provide theory guidance for experiments on antioxidant proteins. However, this predictor cannot be used to design antioxidant proteins through multiple pathways including inactivation of reactive oxygen species, scavenging free radicals, chelation of prooxidative transition metals, reduction of hydroperoxides, and alteration of the physical properties.

### 3.7 Performance Comparisons with the Existing Methods

To evaluate the prediction performance objectively, we compare our method with reference [22] on the same training dataset. Table 4 reports the detailed prediction results obtained by our ensemble classifier and [22] using 10-fold cross validation. From Table 4, our ensemble classifier obtains satisfactory performance and outperforms the method in reference [22]. The sensitivity, specificity, accuracy, MCC, and AUC obtained by the proposed method are about 4%, 4%, 4%, 8% and 3.8% higher than those achieved by the method in reference [22], respectively.

**Table 4:** Performance comparisons of our ensemble classifier with an existing method on the same training dataset.

Method	Sn	Sp	Acc	MCC	AUC
[22]	0.91	0.89	0.90	0.80	0.94
This study	0.95	0.93	0.94	0.880	0.978

To further assess the prediction performance of the proposed method, we make comparisons with [21, 22] on the same independent testing dataset. The performance comparison based on the same dataset is much more reliable, which can reflect the performance of a predictor more objectively. As listed in Table 5, the prediction results of our ensemble classifier are significantly better than those of the method in reference [21]. Although the sensitivity yielded by the method in reference [22] is a little higher than that obtained by our predictor, the specificity, accuracy, and MCC of our method are significantly higher than those achieved by the method in reference [22], which indicates that an unreasonable balance between sensitivity and specificity exists in the method in reference [22]. Our method achieves a balanced performance with a sensitivity of 0.878 and a specificity of 0.860, which is also reflected by an MCC of 0.617. It also gives a satisfactory discrimination power expressed by an accuracy of 0.863 and an AUC of 0.948. From Tables 4 and 5, the predictions deteriorate significantly in the independent testing dataset. This phenomenon may be due to the fact that the independent test set is not used in the learning process of our proposed predictor. The parameters of our proposed predictor are determined in the learning process based on the training dataset. Therefore, the proposed ensemble classifier has fairly good performance in predicting antioxidant proteins, superior to previous methods, which may be conducive to better understanding physiological processes of certain types of diseases and developing novel antioxidation-based drugs.

**Table 5:** Performance comparisons of our ensemble classifier with existing methods on the same independent testing dataset.

Method	Sn	Sp	Acc	MCC	AUC
[21]	0.77	0.77	0.77	0.43	0.83
[22]	0.91	0.79	0.81	0.54	0.94
This study	0.878	0.860	0.863	0.617	0.948

### 3.8 Online Web Server

Since user-friendly and publicly accessible web-servers represent the future direction for developing more practically predictors, we have established a free web-server at http://antioxidant.weka.cc for the method presented in this paper. Users can enter query protein sequences in FASTA format or input the UniProtKB ID of the query protein sequences in the text box area for prediction. When protein sequences are submitted to the server, a job ID is presented to users. The predicted result page will return the input information and predicted result.

## 4 Conclusions

In this study, we have proposed an ensemble predictor using hybrid features extracted from SSI, PSSM, RSA, and CTD to predict antioxidant proteins. We investigate prediction capabilities of various base classifiers and obtain a classifier list based on the accuracy. Based on the ranked classifier list, the idea of IFS is employed to determine an optimal classifier subset. Compared with its component base classifiers, the optimal ensemble classifier achieves much better prediction performance. To improve prediction capability of the model and economize computational time, the Relief-IFS method is adopted to obtain the optimal features. The ensemble method with feature selection is determined as the final predictor for antioxidant protein prediction, which achieves a sensitivity of 0.95, a specificity of 0.93, an accuracy of 0.94, an MCC of 0.880, and an AUC of 0.978. To evaluate the prediction performance objectively, the proposed method is compared with existing methods on the same independent testing dataset. Our method obtains the best specificity of 0.860, accuracy of 0.863, MCC of 0.617, and AUC of 0.948. In addition, our method achieves more balanced performance with a sensitivity of 0.878 and a specificity of 0.860. It is convinced that the proposed ensemble predictor is quite promising in predicting antioxidant proteins.

## Supporting Information

S1 Table The benchmark dataset. The benchmark dataset contains a training dataset and an independent testing dataset. The training dataset is composed of 100 antioxidant and 100 non-antioxidant proteins. The independent testing dataset consists of 74 antioxidant and 392 non-antioxidant proteins.
(XLSX)

S2 Table The ranked feature list given by the Relief algorithm. Within the list, a feature with a smaller index represents a more important one for antioxidant protein prediction. Such a list of ranked features are used to establish the optimal feature set in the IFS procedure.
(XLSX)

S3 Table The Incremental Feature Selection (IFS) results. In the IFS procedures, adding the ranked features one by one, individual predictors for all the feature subsets are constructed using our ensemble classifier and evaluated by 10-fold cross validation. The IFS results are obtained.
(XLSX)
